# Gut Associated Lymphoid Tissue (GALT) primary cells and stable cell lines as predictive models for intestinal health in rainbow trout (*Oncorhynchus mykiss*)

**DOI:** 10.3389/fimmu.2022.1023235

**Published:** 2022-10-20

**Authors:** D. Porter, David Peggs, C. McGurk, Samuel A. M. Martin

**Affiliations:** ^1^ Scottish Fish Immunology Research Centre, School of Biological Sciences, University of Aberdeen, Aberdeen, United Kingdom; ^2^ Skretting Aquaculture Innovation, Stavanger, Norway

**Keywords:** GALT, gut associated lymphoid tissue, beta glucan, rainbow trout, immune, functional feed

## Abstract

The use of functional feeds for farmed fish is now regarded as a key factor in improving fish health and performance against infectious disease. However, the mechanisms by which these nutritional components modulate the immune response are not fully understood. The present study was undertaken to identify the suitability of both primary gut-associated lymphoid tissue (GALT) leucocyte cells and established rainbow trout cell lines as potential alternative methods to test functional feed ingredients prior to full fish feeding trials that can take months to complete. In addition to the primary GALT culture cells, the two rainbow cell lines RTS11 and RTgutGC which are from macrophage and gut epithelial cells, respectively. The cells were stimulated with a variety of pathogen associated molecular patterns (PAMPs) (PHA and Poly I:C) and recombinant rainbow trout IL-1β (rIL-1β), a proinflammatory cytokine, additionally two forms of β-glucan, a prebiotic commonly used aquafeeds were used as stimulants. From this, the suitability of cell models as a health screen for functional feeds was assessed. GALT leucocytes were deemed most effective to act as a health screen over the 4hr time point demonstrating responses to Poly I:C, PHA, and rIL-1β. RTS11 and RTgutGC also responded to the stimulants but did not give a strong T-cell response, most likely reflecting the nature of the cell type as opposed to the mixed cell populations from the primary GALT cell cultures. When stimulated with both forms of β-glucan, GALT leucocytes demonstrated a strong proinflammatory and T-cell response.

## 1 Introduction

Aquaculture faces several important challenges, from the global demand for greater production and the health challenges that accompany increased production, to the challenges associated with the need to identify new ingredients and additives in line with global sustainability standards.

Infectious diseases are the largest cause of economic loss in the aquaculture industry and are controlled by a variety of methods including vaccination, selective breeding, biosecurity, and nutritional intervention (1–3). The use of therapeutics such as antibiotics, where vaccines and other approaches are unable to control disease, can lead to an increase in antibiotic resistance in pathogenic species (4, 5). To improve the robustness of the fish in aquaculture, functional feeds have been developed which may contain immunostimulants, prebiotics, probiotics, and other compounds such as key vitamins and minerals to promote fish health. These dietary components interact within the gut at the interface between nutrition, microbiome, and the immune system (6, 7). Subsequently, modulation of the immune system can occur either through direct interaction with immune cells *via* specific receptors, or through metabolites produced by the intestinal microbial communities. This modulation can lead to improved fish health and increased resistance against disease leading to a reduction in mortalities, recovery time and the use of chemotherapeutic treatments (8). However, the mechanisms by which these functional ingredients interact with the immune system is poorly understood with further research and development of assays to measure immune modulation needed (9).

β-glucans are a commonly used prebiotic/immunostimulant in functional aquaculture nutrition, they are composed of polymers of repeating units of D-glucose linked by β-glycosidic bonds and have many branched side chains (10). β-glucans are naturally occurring components of the yeast and certain algae cell walls (11). Many studies, across different fish species, demonstrate that β-glucans can modulate the immune response triggering various immune pathways including complement, anti-viral and proinflammatory signaling whilst also promoting survival against bacterial and viral pathogens in salmonids (12–15). The health promoting activity of β-glucan based functional feed to the viral pathogen Viral Haemorrhagic Septicaemia Virus (VHSV) was demonstrated with the Skretting Protec™ diet where survival against VHSV was increased in rainbow trout fed the functional feed compared to control diet (15). This diet contains several dietary additives including β-glucans, vitamin E, vitamin C and zinc and resulted in increased magnitude of expression of both immunoglobulins (*IgM, IgT and IgD*) and anti-viral genes including*, MX Dynamin Like GTPase 1* (*MX*)*, and Interferon-gamma* (*IFN-γ*) following infection in comparison to those fish fed the control diet. Many other studies have described the upregulation of key proinflammatory cytokines *Interleukin 1β* (*IL-1β*)*, Tumor Necrosis Factor-alpha* (*TNFα*) and *Cyclooxygenase-2* (*COX-2*) after supplementation with β-glucans which appears to increase resistance to bacterial pathogens (13, 15). The immunostimulatory effects can be considered tissue and species-dependent with the head kidney and spleen showing differing upregulation of inflammatory cytokines in trout (13). To highlight the species differences, in carp, the intestinal response was a decrease in the expression of several inflammatory cytokines (16), possibly reflecting the different trials and challenges performed between research groups, highlighting the complexities of the responses during trials. In carp, the direct response to β-glucans indicates the involvement of C-type lectins (CLEC4C) in the recognition of β-glucan molecules following whole transcriptome analysis by RNA-seq (17). In trout, supplementation with β-glucans showed an upregulation of T-cell activation, both the classical and alternative complement pathways, proinflammatory responses, through genes such as *IL-1β* and *TNFα* and signaling pathways including the P13k-AKT and mTOR signaling pathways (18). Subsequently, an assay to study the effects of β-glucans and other dietary stimulants is needed to categorize responses in different tissues and to identify tolerance to pathogens.

Feeding trials are the gold standard in aquaculture nutrigenomics and involve testing novel ingredients with various parameters; whole tissue histology, performance-based metrics, and transcriptomics/proteomics. However, due to feeding trials using many fish to study just one ingredient, complementary methods have since been developed to identify the mechanisms of action of functional ingredients, which could be especially useful at the early stages of characterization of new products. To examine direct tissue responses several techniques have been developed using explant (19), primary cell cultures (20) or cell lines (21).

In rainbow trout, two cell lines; RTgutGC, an intestinal epithelial cell line (22) and RTS11, a spleen monocyte-macrophage cell line (23) may help explain immunological responses to nutrients. RTgutGC has been used as a health screen for model functional ingredients (24) where nucleotides, mannanoligosaccharides (MOS), and β-glucans were used to identify immunostimulatory effects and intestinal cell barrier function. The RTS11 cells are highly immunologically reactive and have been used extensively in immune function studies (25–27).

Cell lines classically comprise one (clonal) cell type so will not reflect the complexity of the host immune response due to the singular cellular phenotype. To overcome the lack of phenotypes present, primary immune cell cultures from the gut associated leucocyte tissue (GALT) may be a complementary approach for testing functional ingredients as described for rainbow trout (20) and gilthead seabream (*Sparus aurata*) (28). For rainbow trout, flow cytometry and targeted gene expression have been used to identify specific cellular markers which indicate the presence of T-cells, B-cells, and dendritic cells in primary GALT cultures (20) suggesting this may be a suitable model for testing functional feed components.

The aims of the current study were to develop a primary cell culture model that can be used to assay nutritional ingredients for functional feeds. To assess the suitability of the GALT leucocyte assay comparisons between established cell models for the identification of immune responses in the intestine, the permanent cell lines RTS11 and RTgutGC were used. The data generated using GALT leucocytes and rainbow trout cell lines help to further elucidate the mechanisms by which β-glucans act as immunostimulatory molecules.

## 2 Materials and methods

### 2.1 Fish

Rainbow trout (400-500g) were maintained in 250L 1 m-diameter fiberglass tanks with recirculating freshwater at 14°C. Fish were fed twice a day with a commercial diet (Skretting Elite FR 6mm) at 1.5% bodyweight per day and sampled at the same time of day on each occasion used. Fish were killed by schedule 1 method in accordance with the UK Animals (Scientific Procedures) Act, 1986 and associated guidelines, EU Directive 2010/63/EU for animal experiments.

### 2.2 Stable cell line culture

The RTS11 cell line was cultured in flasks (75 cm^2^) at 20°C in growth media (Leibovitz L-15 media + 30% FBS + 1% Penicillin/Streptomycin), the cells were generally non-adherent to the flasks prior to any stimulation. Cells were collected and pelleted by centrifugation at 500g for 10 mins at 4°C before being resuspended in fresh stimulation media (Leibovitz L-15 media + 1% FBS + 1% Penicillin/streptomycin) and adjusted to 5x10^5^ cells/ml, then 1 ml was added to 24 well plates. RTgutGC cells were cultured in flasks at 20°C in growth media (Leibovitz L-15 media (Gibco) + 10% FBS + 1% Penicillin/Streptomycin (1% P/S) (Gibco)). Prior to stimulation, RTgutGC cells were trypsinised, once separated from the flask, cells were resuspended in growth media, washed, and plated as described for RTS11. These cells were then immunostimulated as described below in section 2.4.

### 2.3 Isolation of primary GALT leucocytes

GALT cells were isolated according to the protocol by Attaya et al. (20) with modifications described here. Fish were starved for 24hrs before sampling to reduce the gut contents. In total 24 fish were euthanized for the isolation of GALT leucocytes. Immediately following death, the fish were bled by severing the aorta at the gills. The fish was opened from the ventral side and the hind gut was excised (approx. 2-4 cm in length and ~1g in weight) and placed in 1x PBS on ice (Sigma). The hind gut was rinsed in PBS three times, then cut with a sharp scalpel longitudinally to 0.5-1 cm^2^ squares to aid in rinsing. Gut segments were then placed in 30 ml of PBS in 50 ml falcon tubes before being washed for 20 mins at 40 rpm on an orbital shaker. Gut segments were washed one final time in PBS before being added to 50 ml falcon tubes containing 25 ml of predigestion solution (HBSS (Gibco, 14025092) + 0.145 mg/ml DTT (Sigma, D9779) + 0.37 mg/ml EDTA (Fisher, D/0700/53)). Gut segments were washed in predigestion solution for 20 mins at 40 rpm in an orbital shaker. The supernatant (S1) was then filtered into a 50 ml falcon tube using 100 µm nylon cell strainers (Greiner). S1 was washed using PBS and pelleted twice for 5 mins at 500g at 4°C and the supernatant was discarded, cells were then resuspended in 20 ml of growth media (Leibovitz L-15 media (Gibco) + 10% FBS + 1% P/S) and stored in a 20°C incubator. The gut segments were rinsed using washing medium (HBSS + 0.05 mg DNase1/ml (Sigma, DN25) + 5% FBS + 1% Penicillin/Streptomycin) to remove EDTA and DTT before being placed into the digestion solution (washing media + 0.37 mg collagenase IV/ml (Gibco, 17104-019)) for 2 hours on an orbital shaker at 40 rpm. The supernatant from the digestion was then strained through a 100 µm nylon cell strainer into the tube containing the resuspended S1 phase prior to being washed using PBS with 1% P/S. The supernatant was washed three times using PBS + 1% P/S to remove any collagenase, with the supernatant being discarded. The cells were then resuspended in 5 ml of growth media as described above. The cell suspension was then slowly layered on top of a discontinuous percoll gradient (25% and 75%) (GE healthcare, 17-0891-01) and centrifuged for 30 mins at 400g, at 4°C. Cells were then collected from the interface between the 25 and 75% percoll gradients and washed twice as above. Cells were then suspended in stimulation media before being adjusted to 2.5x10^5^ cells/ml before one ml was added to 24 well plates to which stimulants were added as described in 2.4.

### 2.4 Immune stimulation of cell lines and primary GALT cells

Five different stimuli were used; 100 µg/ml Poly I:C (Sigma, P1530), 10 µg/ml PHA (Sigma, 61764), 20 µgml^-1^ recombinant IL-1β [Provided by Dr. Tiehui Wang (Scottish Fish Immunology Research Centre)] (29), and 100 µg/ml of two molecular forms of β-glucan termed M and F (provided by Skretting AI, Norway). M is M-glucans (Biotec), whilst F is Fibosel (Trouw). The two glucans differ in purification but both are in particulate form and are not highly soluble.

Stock solutions for Poly I:C and PHA were made in L-15 media with aliquots stored in -20°C until use. rIL-1β stock solution was stored in -80°C, and aliquots were diluted in L-15 media when in use. Fresh M-glucans and Fibosel stock solutions were made from a powdered version of each, suspended in L-15 media, and stored at 4°C until use. Stock solutions were then added to wells containing cells diluted in media to give the working solutions. Stimulants were added immediately after plating. All experiments were performed in randomized design with quadruplicate wells that were treated independently through the entire protocol (n = 4).

### 2.5 GALT leucocyte viability

GALT leucocytes were suspended in growth media and incubated in 24 well plates at 5x10^5^ cells/well in 1ml of stimulation media. These cells were incubated at 20°C for 4 hrs and 24 hrs, where they were counted using a Neubauer chamber with 0.5% trypan blue. The viability was determined using the 0 hr time point as controls.

### 2.6 Transcriptional analysis

RNA was extracted from cells in individual wells (2.5x10^5^ in the case of GALT Leucocytes and 5x10^5^ for RTS11 and RTGutGC) using 750µL of TRI Reagent (Sigma) in accordance with the manufacturer’s instructions. The RNA pellet was washed using 80% ethanol, dissolved in RNase-free water, and stored at -80°C until use. Quality control of the samples was determined using a Nanodrop spectrophometer and the integrity of the RNA was assessed by an Agilent Bioanalyzer 2100. RNA (500 ng) was used as template for reverse transcription and generation of cDNA using the Qiagen QuantiTect Reverse Transcription Kit following manufacturer’s instructions. The cDNA was diluted 10x with RNase-free water and stored at −20°C until use.

Gene expression was determined for proinflammatory cytokines (*IL-1β, Interleukin-8* (*IL8*)), an anti-inflammatory cytokine*, Interleukin-10* (*IL-10*), a well characterized antimicrobial peptide, *Hepcidin antimicrobial peptide* (*HAMP*) and an acute phase reactant, *serum amyloid-alpha* (*SAA*), T-cell markers *Interleukin-4/13* (*IL-4/13*) and *IFN-γ* and anti-viral marker genes *MX and Interferon-1 alpha (IFN-1α)* by qPCR (**Table 1**). Amplification was performed using Agilent Brilliant III Ultra-Fast SYBR and amplification were run on a Roche Lightcycler 480 machine, PCR cycles were: initial denaturation of 3mins at 95°C followed by amplification by 40 cycles (5s at 95°C, 10s at 60°C and 1s at 72°C). The expression of target genes was normalized to the relative expression of the mean of three housekeeping genes *Elongation Factor-1 alpha (ELF-1α), Ribosomal Protein L4 (RPL4)* and *Ribosomal Protein S29 (RPS29)*. Three housekeeping genes were used to minimize random errors involved with qPCR. The gene expression was calculated using the Genex 5 software (Multid) to generate relative gene expression which was then used to calculate fold change in comparison to the control samples.

**Table 1:** Primers used for qPCR analysis.

Gene	Forward Primer (5’-3’)	Reverse Primer (5’-3’)	Product Size (bp)	Accession Number	Source
EF1-α	CAAGGATATCCGTCGTGGCA	ACAGCGAAACGACCAAGAGG	327	AF498320	20
RPL4	CCTTCAGAAACATCCCTGGTATCAC	GGGCAGATTGTAGTCTACCTTGAGAG	182	BT057966	30
RPS13	CCCTCTCAGATCGGTGTGATCC	TCCTTGTCCTTTCTGTTCCTCTCC	191	BT059859	30
RPS29	GGGTCATCAGCAGCTCTATTGG	AGTCCAGCTTAACAAAGCCGATG	167	BT043522	30
MX-1	CGTCCCAGACCTCACACTCATC	TGCCATCTTCAAAGCCTCTGTG	187	OMU30253	31
IFN-1α	GTGTGTCATTGCTGTGACTGGA	TTTGTGATATCTCCTCCCATCTG	95	AJ580911	31
SAA	AGTCATCAGTAATGGCCGGGA	AAAAGCTTGTTTGGAATTTGGTCCT	205	NM_001124436	
HAMP	AGTCCCTCATCCGCTGACAT	CAAATAGCGGCGCTCTCCAAT	93	HQ711993.1	
IFN-γ	GTAGCCTGCCGTTTTGAGCA	TGACGGGAGGAGGAACGTAA	250	NM_001124416.2	
Il-4/13β1	GAGATTCATCTACTGCAGAGGATCATGA	GCAGTTGGAAGGGTGAAGCTTATTGTA	255	HG794522	20
TNFα2	CTGTGTGGCGTTCTCTTAATAGCAGCTT	CATTCCGTCCTGCATCGTTGC	99	AJ401377	20
IL-8	GAAACTCGCCACAGACAGAGAA	AGTGTGTTGTTATCTCGCTGGTAA	114	HG917307.1	
IL-10	ACATCCCTGCTGGACGAAGG	GGCAGCACCGTGTCGAGATA	101	NM_001245099.1	
IL-1β	CTGCACCTAGAGGAGGTTGCG	GAAACGCACCATGTCGCTCT	72	NM_001124347.2	

### 2.7 Statistical analysis

Data handling, calculation of fold change and statistical analysis of relative expression using a two tailed students T-test, were performed in Microsoft Excel 2016. Statistical analyses of the fold changes for RTS11 time course experiments were carried out using two-tailed Anova in R. Graphical representation of the data was performed in GraphPad Prizm 5.

## 3 Results

### 3.1 GALT leucocyte viability

GALT leucocytes had a viability of 95.52% at 4hrs and 53.1% at 24hours following extraction compared to 0hrs. This matches GALT leucocyte viability previously reported by Attaya et al. (20) observed a decrease from 93% at 4hrs to 53% at 24hrs. As a result of this, all experiments were performed at the 4hrs time point to remove any artifacts of cell death and related responses. GALT leucocyte extractions were carried out separately for each fish to ensure no non-self-recognition and subsequent modulation of the immune system, to ensure the effects seen were only in response to PAMPs.

### 3.2 Comparisons of gene expression in RTS11, RTgutGC and primary GALT cell in response to Poly I:C stimulations

**Figure 1:**
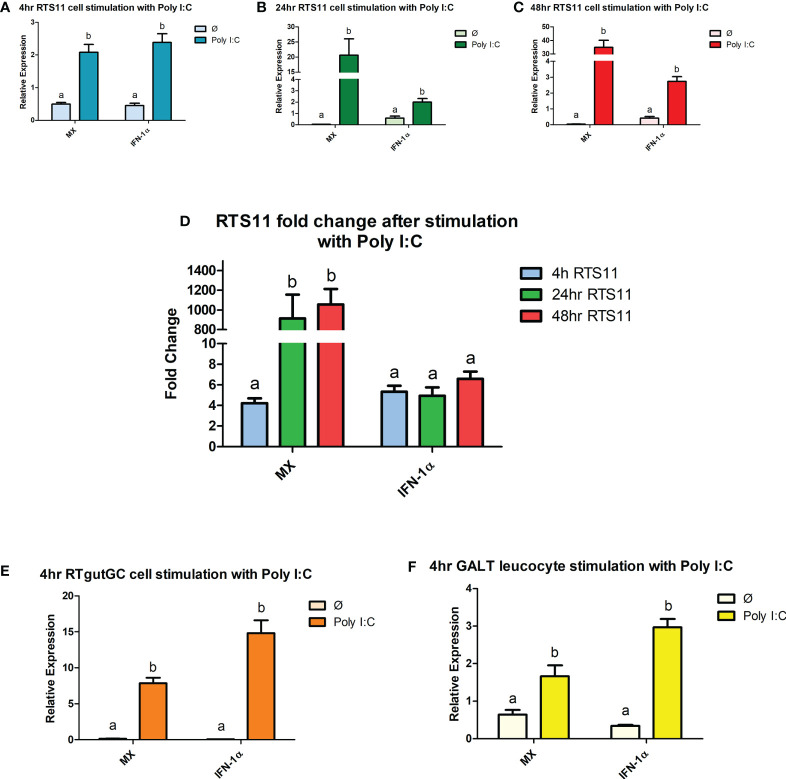
RTS11, RTgutGC and GALT leucocyte response to Poly IC. **(A)** RTS11 4hr Poly I:C stimulation showing relative expression. **(B)** RTS11 24hr Poly I:C stimulation showing relative expression. **(C)** RTS11 48hr Poly I:C stimulation showing relative expression. **(D)** RTS11 Fold Change between time points (4, 24 and 48 hr). **(E)** RTgutGC 4hr Poly I:C stimulation showing relative expression. **(F)** GALT leucocyte 4hr Poly I:C stimulation showing relative expression. For relative expression samples were compared between stimulated and unstimulated samples. ^a^ and ^b^ denote a significant difference where p < 0.05.

To examine the capacity for mounting an interferon/antiviral response, Poly I:C was used as a PAMP as it is an effective inducer of the type I interferon response. At 4hrs following stimulation all the cells showed a significant increase in IFN-1a following the poly I:C stimulation (RTS11 4.2-fold, RTgut 57-fold, GALT 2.92-fold) (**Figures 1A–F**). The RTS11 were also sampled at 24 and 48hr but there was no significant increase in *IFN-1a* at the later time points. The RTgutGC cells showed the greatest magnitude of response with a ~200-fold increase in *IFN-1α*, whereas the GALT cells showed a ~9-fold increase (not shown in figure). The interferon response gene and *MX* were significantly increased in all the poly I:C stimulated cells confirming the response in these cells at 4hrs post stimulation compared to non-stimulated control cells. The RTgutGC showed the greatest increase in *MX* at 4hrs with an approx. 200-fold increase. At 24 and 48hr the RTS11 cells showed high expression of *MX* compared to the control. These results confirm that GALT leucocytes respond to this viral mimic and give similar responses to the macrophage cell line at the 4hr time point. The background expression of both *MX* and *IFN-1α* is slightly higher in GALT cells compared to RTgutGC and RTS11 hence the fold change expression may appear lower.

### 3.3 Gene expression in RTS11, RTgutGC and GALT leucocytes in response to PHA stimulation

**Figure 2:**
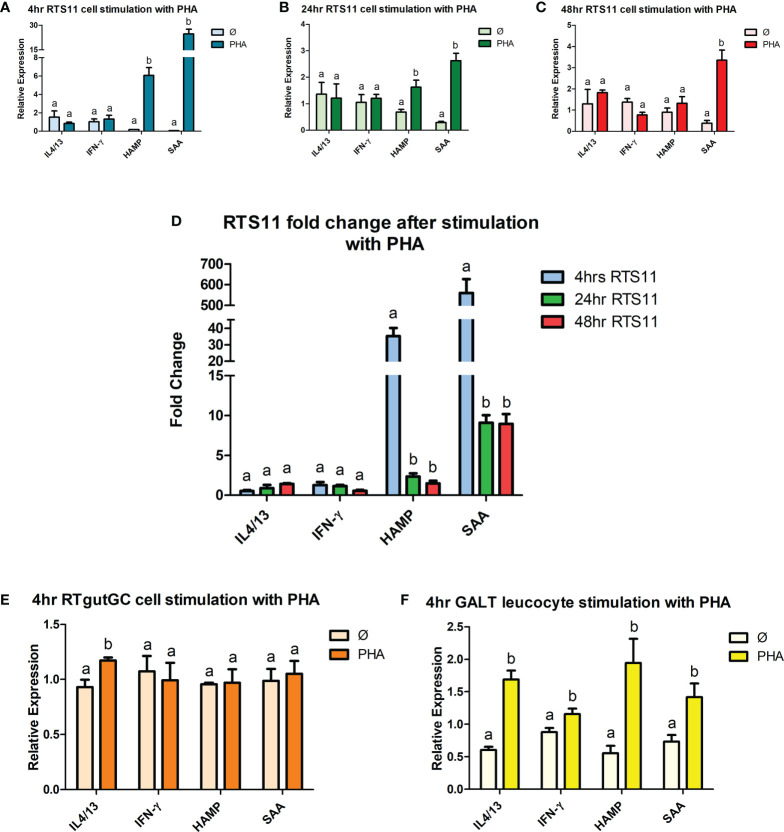
RTS11, RTgutGC and GALT leucocyte response to PHA. **(A)** RTS11 4hr PHA stimulation showing relative expression. **(B)** RTS11 24hr PHA stimulation showing relative expression. **(C)** RTS11 48hr PHA stimulation showing relative expression. **(D)** RTS11 Fold Change between time points (4, 24 and 48 hr). **(E)** RTgutGC 4hr PHA stimulation showing relative expression. **(F)** GALT leucocyte 4hr PHA stimulation showing relative expression. *For relative expression samples were compared between stimulated and unstimulated samples.* ^a^ and ^b^ denote a significant difference where p < 0.05.

PHA is a potent stimulator of the proinflammatory response and induces differentiation of T cells. The response to the PHA in the GALT cells revealed a small but significant increase of expression to *IL-4/13* and IFN-γ (**Figure 2F**), the markers for inflammation, SAA and HAMP were also induced showing both T cell and inflammatory response. The RTgutGC cell line had a minimal response to the PHA stimulant, with only *IL-4/13* showing a difference in expression (**Figure 2E**). However, in the RTS11 cell line, significant responses were only observed for *SAA* and *HAMP* at both 4hrs and 24hrs post stimulation suggesting the proinflammatory response was being upregulated but not the T-cell response (**Figures 2A–D**). RTS11 is a macrophage cell line and as a result the T-cell mediated responses were not found to be altered except with *IFN-γ* being significantly decreased at 48hrs.

### 3.4 Gene expression responses in RTS11, RTgutGC and GALT leucocytes to recombinant trout IL-1β

**Figure 3:**
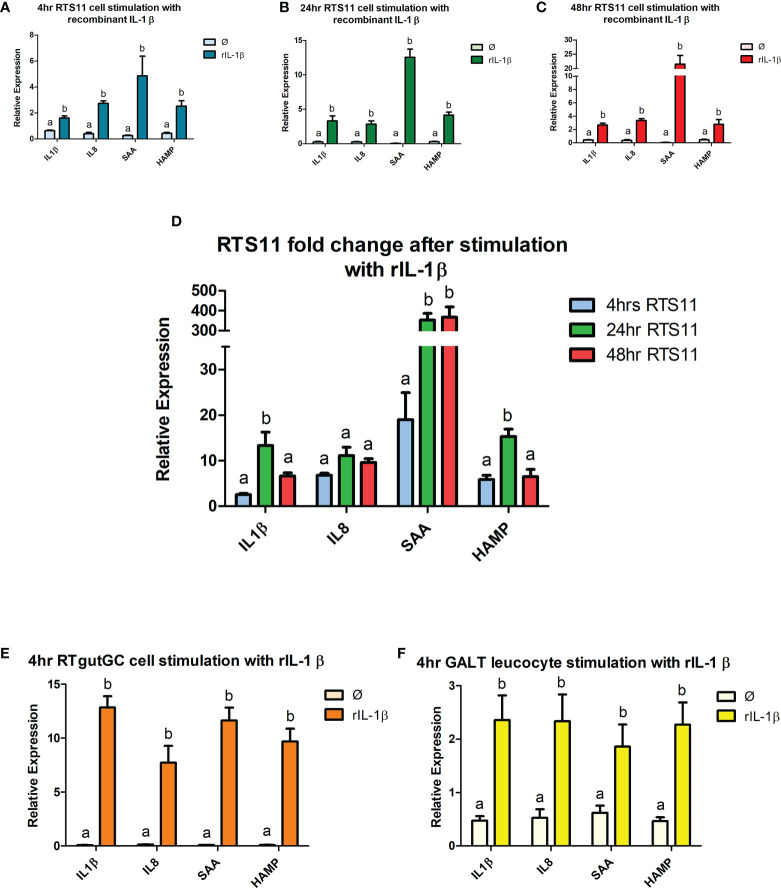
RTS11, RTgutGC and GALT leucocyte response to rIL-1β. **(A)** RTS11 4hr rIL-1β stimulation showing relative expression. **(B)** RTS11 24hr rIL-1β stimulation showing relative expression. **(C)** RTS11 48hr rIL-1β stimulation showing relative expression. **(D)** RTS11 Fold Change between time points (4, 24 and 48 hr). **(E)** RTgutGC 4hr rIL-1β stimulation showing relative expression. **(F)** GALT leucocyte 4hr rIL-1β stimulation showing relative expression. For relative expression samples were compared between stimulated and unstimulated samples. ^a^ and ^b^ denote a significant difference where p < 0.05.

Recombinant IL-1β is an effective proinflammatory stimulant and drives a high level of gene expression responses. Significant increases in gene expression responses were found in a panel of proinflammatory responding genes, with two cytokines *IL-1β* and *IL-8* and two downstream responding genes, *SAA* and *HAMP*. RTS11, RTgutGC and GALT leucocytes all responded to rIL-1β stimulation (**Figures 3A–F**). RTS11 showed a significant increase at all time points to *IL-1β, IL-8, SAA*, and *HAMP* with higher responses found at 24 and 48h, especially for SAA, whereas *HAMP* was highest at 24hr and then the magnitude of response dropping at 48hr (**Figures 3A–D**). The RTgutGC showed the largest expression responses to the rIL-1β after 4hrs in all genes tested, with the GALT leucocytes also exhibiting significant changes at the 4hr time point in *IL-1β, IL-8, SAA*, and *HAMP*, but at a lower magnitude to that of the RTgutGC cells. These results further demonstrate GALT leucocytes’ ability to respond to a proinflammatory stimulus as previously demonstrated by PHA. GALT leucocytes show similar responses to the RTS11 cell line suggesting that the responses are being driven by the macrophage response at the 4hr time point.

### 3.5 Gene expression responses to two β-glucans in cell lines and GALT primary leucocyte cultures

β-glucans are regarded as both prebiotics and as PAMPS, however the precise mode of function of the molecules is not known. Different β-glucan formulations may invoke different responses in cells. Here two different β-glucans are examined for the direct response on two cell lines, a macrophage cell line RTS11, a gut derived cell line RTgutGC and primary GALT leucocytes.

#### 3.5.1 Gene expression responses to M-glucans

**Figure 4:**
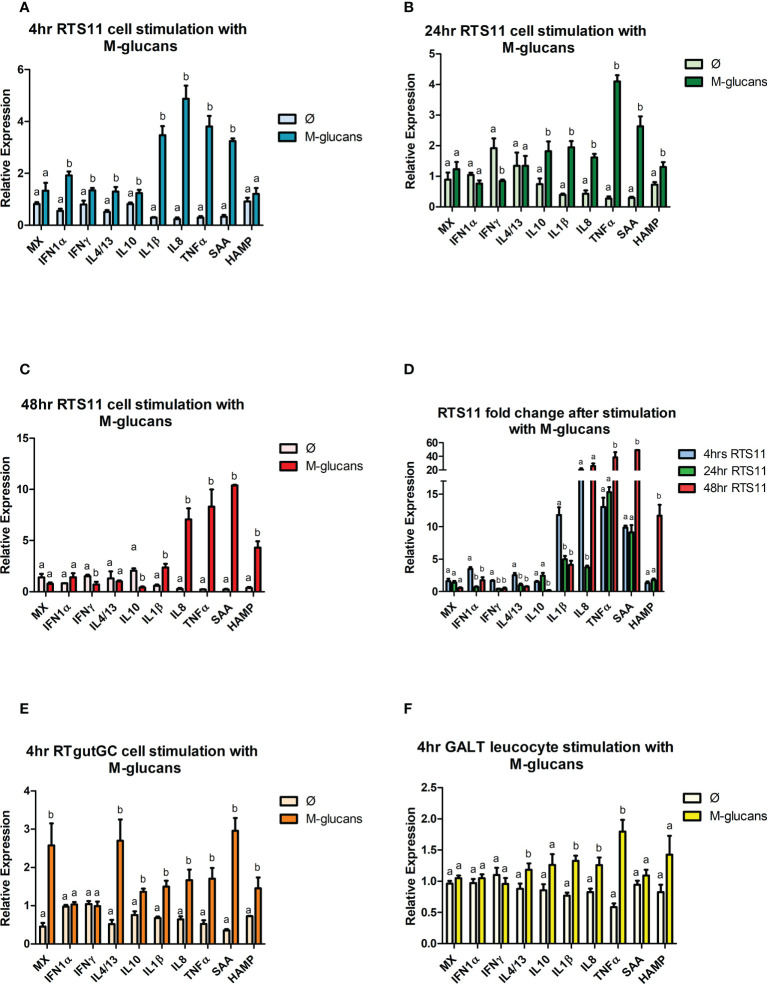
RTS11, RTgut and GALT leucocyte response to M-glucans. **(A)** RTS11 4hr M-glucans stimulation showing relative expression. **(B)** RTS11 24hr M-glucans stimulation showing relative expression. **(C)** RTS11 48hr M-glucans stimulation showing relative expression. **(D)** RTS11 Fold Change between time points (4, 24 and 48 hr). **(E)** RTgutGC 4hr M-glucans stimulation showing relative expression. **(F)** GALT leucocyte 4hr M-glucans stimulation showing relative expression. For relative expression samples were compared between stimulated and unstimulated samples. ^a^ and ^b^ denote a significant difference where p < 0.05.

The cells that were exposed to M-glucans showed significant increases in gene expression for the proinflammatory marker genes *IL-1β, IL-8*, and *TNFα* in all three cell types (**Figure 4**). The magnitude of response is highest in the RTS11 cells (**Figures 4A–C**), which further increases at the later time points for this cell line at 24 and 48h. Secondary responding gene *SAA* indicated a small but significant response at the 4hr time point in the RTS11 cells. At 48hrs post stimulation RTS11 showed significant increases in the responses of both secondary responding genes (*HAMP* and *SAA*). In the RTgutGC cells the proinflammatory cytokines *IL-1β, IL-8* and *TNFα* were all increased significantly as was *SAA* (**Figure 4E**), a similar response was observed in the GALT cells where *IL-1β, IL-8* and *TNFα* were all increased significantly (**Figure 4F**). The anti-inflammatory cytokine, *IL-10*, was found to be significantly increased in the RTgutGC cells and RTS11 at 4hrs but not in the primary GALT cells.

There is minimal evidence for an antiviral gene expression response in the RTS11 with only IFN-α being significantly increased at 4h, but the expression was not found during the later time points, the MX gene was not increased here suggesting the M-glucans in the RTS11 did not behave like a viral PAMP (**Figure 4D**). In the RTgutGC cells a significant rise in MX gene expression was observed, whereas the GALT cells showed no increase in either of these genes. M-glucans may also modulate the T-Cell response with *IL-4/13* being significantly upregulated across all cell types at the 4hr time point, with *IFN-γ* also increased in the RTS11 cells.

#### 3.5.2 Gene expression responses to Fibosel

**Figure 5:**
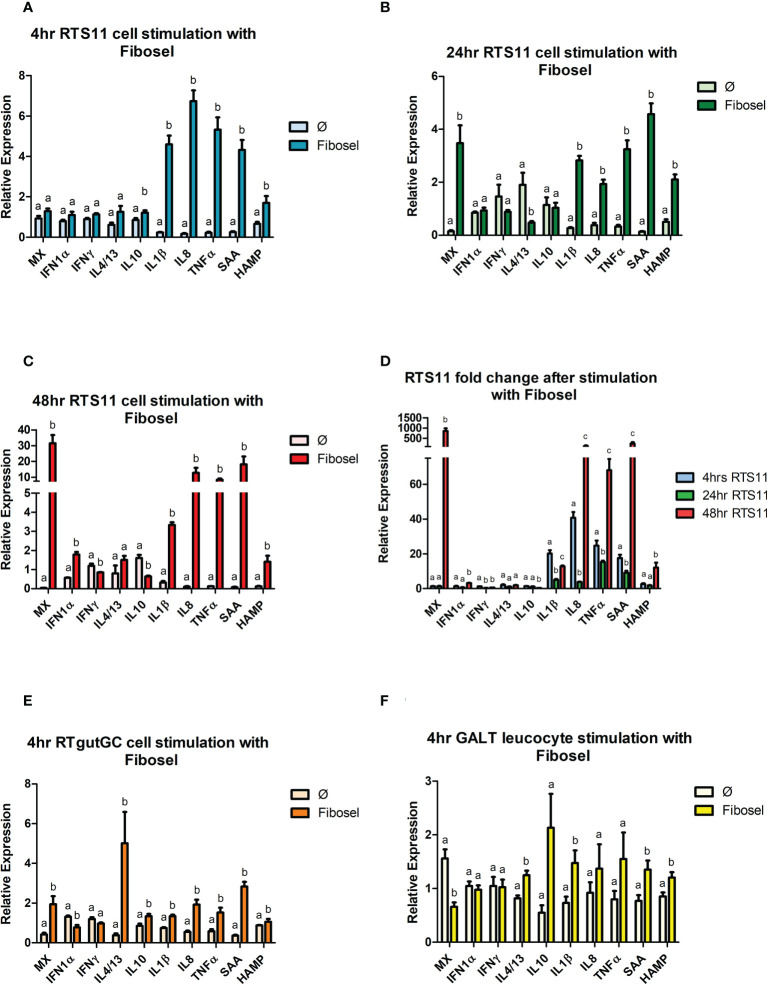
RTS11, RTgut and GALT leucocyte response to Fibosel. **(A)** RTS11 4hr Fibosel stimulation showing relative expression. **(B)** RTS11 24hr rIL1 β stimulation showing relative expression. **(C)** RTS11 48hr Fibosel stimulation showing relative expression. **(D)** RTS11 Fold Change between time points (4, 24 and 48 hr). **(E)** RTgutGC 4hr Fibosel stimulation showing relative expression. **(F)** GALT leucocyte 4hr Fibosel stimulation showing relative expression. For relative expression samples were compared between stimulated and unstimulated samples. ^a,b,^and ^c^ denote a significant difference where p < 0.05.

The three different cell models all indicated a common gene expression response to the Fibosel with *IL-1β*, *SAA* and *HAMP* being significantly upregulated at 4hr post stimulation, the RTS11 cells had the greatest magnitude of response (**Figure 5**). *TNFα* and *IL-8* were significantly upregulated in both RTS11, at every time point, and RTgutGC after 4hrs. Later time points in RTS11 cells showed a sustained response with *IL-8* and *TNFα* peaking at 48hrs (**Figures 5A–D**). *IL-10* was significantly upregulated after 4hrs in both RTS11 and RTgutGC. *IL-10* expression showed no significant change at the 24hr time point and was significantly downregulated at the 48hr time point in RTS11 cells. GALT cells showed non-significant increases in expression of *IL-10*. The expression of the secondary proinflammatory responders *SAA* and *HAMP* were both significantly increased in GALT, RTgutGC and RTS11 cells with the response increasing with time post challenge. Interestingly, the proinflammatory markers (*IL-1β, IL-8, TNFα, SAA*, and *HAMP*) all demonstrated a “U-shaped response” over the time course with RTS11 cells (**Figure 5D**). The gene expression response related to antiviral activity showed mixed responses between the cell types. The RTS11 cells responded strongly to Fibosel with the *MX* being significantly increased in expression at 24 and 48h, but not at 4h. The expression of *MX* was also found to be increased in the RTgutGC cells but decreased in expression in the GALT cells. *IFN-1α* showed a minimal change in the RTS11 cell line at all time points with downregulation seen in RTgutGC. *IL-4/13* was found to increase in expression in both the RTgutGC and the GALT cells but not in the RTS11, with *IFN-γ* not responding to the Fibosel in any cell type examined.

#### 3.5.3 Comparison between M-glucans and Fibosel

**Figure 6:**
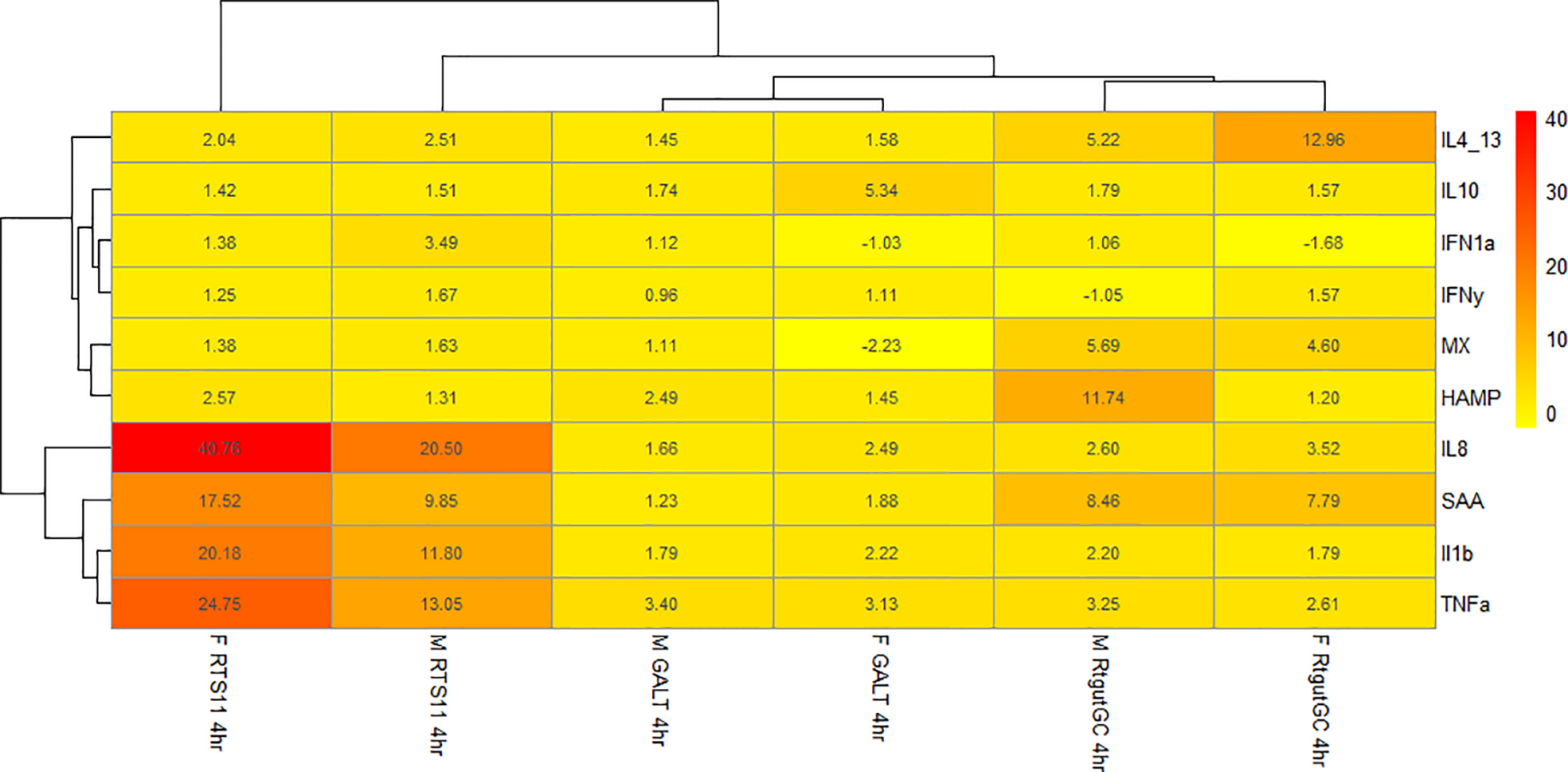
Heatmap of fold change for samples stimulated with either M-glucan or Fibosel across all three cell culture models.

**Figure 6** shows a heatmap of the β-glucan stimulated samples at the 4hr time point. Both Fibosel and M-glucans can drive a proinflammatory response shown by upregulation of *IL8, IL-1β, SAA, TNFα* and *HAMP* in RTS11 cells, this response is also observed to a lesser extent in RTgutGC. This response is also seen in GALT but to a much lower extent in terms of fold change, potentially due to different cell types being present so a single response is not seen and is more subtle. Fibosel can drive the proinflammatory response to a higher extent compared to M-glucans in RTS11 cells and in GALT cells however in RTgutGC it is the other way around. The secondary response gene HAMP also shows unique differences between cell types and stimulants. Both β-glucans can modulate the *HAMP* response with M-glucans driving a larger response in M-glucans in RTgutGC and GALT whilst Fibosel drives a larger response in RTS11. Viral genes show very little change across all cell lines with only *MX* seeming to be upregulated in RTgutGC to a similar extent between both M-glucans and Fibosel. These responses seen may offer an indication into the pathways that are driven by β-glucans, with the proinflammatory response and antibacterial genes seemingly affected more by β-glucan supplementation. Whilst the GALT response is more subtle as previously mentioned levels of background expression were much higher in this cell line in comparison to both RTS11 and RTgutGC so may demonstrate a lower fold change compared to a single cell type’s response and as such, may not be seen as effectively as a cell line.

## 4 Discussion

Both *in-vivo* experiments, and cellular models can be used to identify molecular responses to PAMPs or functional ingredients through the advancements in *‘omic’* technologies (32, 33). Many of the important economic aquaculture species have genomes which are now fully characterized and annotated allowing for in-depth immuno-transcriptomic experiments to illustrate the molecular machinery involved in the immune response (34). *In-vivo* studies have been used to identify the molecular responses to prebiotics and subsequent pathogenic challenges offer an insight into the methods in which prebiotics may modulate the immune response to protect against pathogens. The use of *in-vivo* models highlights the overall response where there is likely to be both direct and in-direct interaction of prebiotics with the GALT tissue. However, the extent to which these responses are being caused by the direct or in-direct interactions of prebiotics are not fully known. On the other hand, cell lines and other cellular models can be used to identify the direct effects of PAMPs and functional ingredients on specific cell types and their subsequent immune response as such these models can be used either as stand-alone models or to complement *in-vivo* studies to offer greater understanding and knowledge of the cellular mechanisms. For this study both the RTS11 and RTgutGC cell lines were used in combination with GALT primary leucocytes to determine how the cells direct immune responses to both characterized PAMPs and two β-glucans that are used as functional feed compounds. These cell culture models have been previously established as viable assays to identify immune responses; RTS11 (35), RTgutGC (24) and GALT leucocytes (20).

The initial experiments performed to assess the different responses of the cell types to PAMPS, secondly the cells were exposed to the two forms of β-glucan to determine the extent of direct cellular stimulation, and if so what type of response these molecules induced. The RTS11 cell line has been extensively characterized for gene expression responses to immunostimulants (24, 35, 36) especially for proinflammatory and antiviral responses (37) and is the go-to cell line for many immunological studies. RTS11 cells are derived from the spleen and are believed to have phenotype representing macrophages (23). RTgutGC was developed by 22, these cells represent epithelial cells. The role of these epithelial cells in immunological responses is relatively unknown. Recent studies by Alkie et al. (38) have reported immunological responses in the RTgutGC cells in response to Poly I:C showing an increase in several genes involved in antiviral activity including *IFN-1α*, *MX-1* and *VHSV-induced gene 3 (Vig3)* at both 6 and 12 h post stimulation. Both cell lines are believed to represent a single cellular phenotype, whereas the GALT primary cells may represent a diverse collection of leucocytes as previously described (20). Some cell lines (RTgutGC and RTgutF) have been used in conjunction with one another to offer a cell line model that represent a closer representation of the gut model and barrier function (39).

Primary cell cultures in contrast to permanent cell lines have the advantage of retaining the diversity of immune cell types from the organ they are extracted from, but there are the added complications of extraction time, and the phenotypic and genetic variability of the animal from which these cells are obtained. In this experiment, we assess how GALT cells respond in comparison to the responses seen in the characterized cell lines of rainbow trout. Primary leucocytes have been isolated from many tissues including isolated head kidney (40), gill (41), blood (42) and have been used to identify immune responses with these tissues being classically regarded as primary and secondary immune organs. However, it is becoming clearer that the intestine and GALT plays a very important and immediate role in immune responses as a consequence of dietary manipulations. Currently there is a limited amount of literature on the isolation of leucocytes from the gut in salmonids (20, 28, 43). GALT leucocytes from the whole gut have been tested against several different PAMPs (20), in this study our aim was to use just the distal intestine due to its theoretical importance as an immunological organ and is the basis of many feeding trial experiments on the gut. Cell viabilities for GALT from the hind gut in this experiment matched previously reported viabilities from GALT cells extracted from the whole gut as shown by 20. Whilst there is good viability at 4hrs the GALT leucocytes show a significant drop after 24hrs probably due to the length of the extraction method used to isolate cells from both the lamina propria and intraepithelial layers of the gut. To our knowledge, this is the first comparison between GALT leucocytes and cell line models. The major difference between cell line models and primary cultures are the number of cellular phenotypes present. Cell line models typically only have one cell type present and as such may only display one or two phenotypes. GALT leucocytes have been demonstrated to contain multiple cell types with key cell markers seen for several different T-, B-cells, macrophages, neutrophils, and dendritic cell populations (20) and display a much larger repertoire of cellular phenotypes. This increase in cell types may offer a larger number of potential cellular responses to be identified in these models.

All cell types responded to the viral mimic Poly I:C and there was a clear increase in expression of both *IFN-1α* and *MX* following 4 hours of stimulation. At 4hrs the magnitude of response was highest in the RTgutGC cells, also of interest the GALT leucocytes had higher background expression so may not have as large a magnitude of response. RTS11 cells were also able to respond to Poly I:C stimulation. This confirms the GALT cells can respond to this viral mimic.

PHA has properties as a strong T-cell stimulant, as well as being able to stimulate proinflammatory mediators. PHA has the capacity to stimulate RTS11 cells with studies showing an increase in expression of *Interferon-gamma inducible protein (γIP)* (37), an IFN-γ responsive gene, suggesting these cells have receptors that enable PHA responses. The T-cell marker genes we chose to explore for PHA responses were IFN-γ and IL-4/13, neither cell lines responded with an increase in expression of these two markers in our experiments. Interestingly, the GALT cells, which are expected to have a diverse repertoire of cell types did respond to PHA with these genes both being significantly increased in expression at 4-hour post stimulation. PHA can also act as a proinflammatory mediator (44) as seen in our study with RTS11 and GALT cells demonstrating a significant upregulation of both *SAA* and *HAMP*. This change was not observed in the RTgutGC cell line suggesting that this epithelial cell line can only respond to certain proinflammatory triggers due to the constrained cell type and receptor repertoire.

IL-1β is a key proinflammatory cytokine involved in many different cell signaling pathways and is often upregulated during the early proinflammatory response. Recombinant IL-1β can trigger the immune response in RTS11 cells with over 20% of genes within this enriched biological process, including genes such as *Hepcidin* and immune response protein 1, being differentially expressed (35). For the rIL-1β response we decided to assess two early phase cytokines *IL-1β* and *IL-8* and two secondary mediators *SAA* and *HAMP.* All four genes assessed showed an increase in response to rIL-1β after 4 hours of stimulation in all cell culture models. Interestingly, the response at 4 hours between RTS11 and GALT were similar for all four genes potentially suggesting the macrophage cells were driving the response to rIL-1β. GALT leucocytes demonstrated higher background levels of expression compared to both RTS11 and RTgutGC samples. This data validates the need for further primary cell culture models to be developed. Gut specific cell culture models are needed to provide a unique insight into the mechanisms behind the interface between nutrition and the immune system (33).

The use of the PAMPS established the potential for the different cell lines and GALT cells to respond to the characterized stimulants, The next question was how these cells respond to the β-glucans. β-glucans can act as an immunostimulant causing the upregulation of the proinflammatory response as demonstrated by previous cell line models using both RTS11 and RTgutGC (24, 45, 46). In the current work, we have used two forms of β-glucans were used to identify if the refinement of the compound has an impact on the immune response to these molecules. Stimulation of RTS11, RTgutGC and GALT leucocytes, showed an increase in the proinflammatory markers tested with *IL-1β, IL-8, TNFα* and *SAA* commonly being significantly upregulated across both M-glucan and Fibosel stimulated samples. This data suggests that β-glucans can modulate the proinflammatory response directly and not just as a prebiotic (*i.e.*, only having effect *via* changes in microbiome activity) (47). Of the two β-glucans tested, M-glucan was able to induce a greater upregulation of the proinflammatory response. Previous research using the M-form (45) are consistent with the results of the present study. This indicates that the particulate β-glucan form acts as a stronger modulator of the proinflammatory response. Both β-glucans were able to significantly increase the *IL-4/13* gene in GALT leucocytes and RTgutGC, however only M-glucans were able to modulate RTS11 cells at 4hrs. Fibosel stimulation resulted in a much larger upregulation of the *IL-4/13* gene after 4 hours compared to M-glucans. These results further validate the idea that β-glucans can act as an immunostimulant interacting directly with immune cells.

Previous *in-vivo* studies have shown β-glucan administration generates a short-lived immune stimulatory effect resulting in enhanced resistance to both viral and bacterial infections (17, 48). Noticeably, short term responses on viral genes on GALT leucocytes had no effect on viral response. A viral response was seen in RTS11 cells treated with fibosel at the 48hr time point with *MX and IFN-1α* being upregulated, this response was not seen in cells treated with M-glucans. Further studies are needed to quantify whether other type 1 interferons are involved. Our data suggests that β-glucans may have immunostimulatory properties more aligned with antibacterial activity than antiviral activity in the short term innate immune response.

There is emerging evidence that β-glucans may be involved in immunotolerance as demonstrated using cell line approaches in trout (45) and primary cell cultures using carp macrophages (17). However, more research is needed to identify the extent to which this immunomodulation offers protection to pathogenic stimulants in both *in-vitro* and *in-vivo* trials and against bacterial or viral pathogens.

In summary, we demonstrate the importance of cellular models that contain multiple phenotypes present to ensure multiple immune cell types’ responses are captured. We have demonstrated the effectiveness of RTS11 and RTgutGC in combination with GALT leucocytes as potential screening methods for functional feed analysis. With RTS11 and GALT leucocytes showing a clear indication of the immune pathways modulated. RTS11 and GALT leucocytes showed good responses against all PAMPs and could act to show relevance against bacterial pathogens and tolerance studies in future studies. Whilst GALT leucocytes showed clear responses to PAMPs and β-glucans, cell viability is still an issue after 24hrs being at 53%, so studies may be limited to 4-8hrs. However, using GALT leucocytes alongside RTS11 cells or an *in-vivo* bacterial challenge would enable tolerance studies to be carried out in conjunction with GALT immune modulation. This study further highlights the action of β-glucans as an immunostimulant with both β-glucans being able to modulate key proinflammatory cytokines and T-cell. Future studies using cell line models or *in-vivo* experiments will further demonstrate if supplementation with β-glucans can offer protection against pathogenic challenges.

## Data availability statement

The original contributions presented in the study are included in the article/supplementary files. Further inquiries can be directed to the corresponding author.

## Ethics statement

Fish were looked after in accordance to UK Animals (Scientific Procedures) Act, 1986 and associated guidelines, EU Directive 2010/63/EU for animal experiments. All animal procedures were carried out under UK project licence PFF8CC5BE. The study was reviewed and approved by the University of Aberdeen Animal Ethics Committee.

## Author contributions

DPo contributed to the design of the experiment, sample acquisition, data analysis, and wrote the manuscript. SM contributed to the design of the experiment, supervision of the project, and reviewed the manuscript. DPe and CM reviewed the manuscript and provided B-glucans. All authors contributed to the article and approved the submitted version.

## Funding

This work was funded by the University of Aberdeen.

## Acknowledgments

This work was funded by Skretting AI and the University of Aberdeen. Technical support by Dr Dawn Shewring was greatly appreciated. We also thank Dr Tiehui Wang, Scottish Fish Immunology Research Centre for providing the rIL-1β.

## Conflict of interest

CM and DPe are employees of Skretting AI.

The remaining authors declare that the research was conducted in the absence of any commercial or financial relationships that could be construed as a potential conflict of interest.

The authors declare that this study received funding from Skretting AI. The funder had the following involvement in the study: review of manuscript and provision of β-glucan samples.

## Publisher’s note

All claims expressed in this article are solely those of the authors and do not necessarily represent those of their affiliated organizations, or those of the publisher, the editors and the reviewers. Any product that may be evaluated in this article, or claim that may be made by its manufacturer, is not guaranteed or endorsed by the publisher.

## Abbreviations

BG, β-glucan; GALT, Gut Associated Lymphoid Tissue; MOS, Mannanoligosaccharides; PAMP, Pathogen associated molecular pattern; SAA, Serum Amyloid A; VHSV, Viral Haemorrhagic Septicaemia Virus.
